# Human γ-Herpesvirus Infection, Tumorigenesis, and Immune Control in Mice with Reconstituted Human Immune System Components

**DOI:** 10.3389/fimmu.2018.00238

**Published:** 2018-02-12

**Authors:** Christian Münz

**Affiliations:** ^1^Viral Immunobiology, Institute of Experimental Immunology, University of Zürich, Zürich, Switzerland

**Keywords:** Epstein–Barr virus, Kaposi sarcoma-associated herpesvirus, natural killer cells, T cells, primary effusion lymphoma, lymphoproliferative disease

## Abstract

The human γ-herpesviruses Epstein–Barr virus (EBV or HHV4) and Kaposi sarcoma-associated herpesvirus (KSHV or HHV8) are each associated with around 2% of all tumors in humans worldwide. However, investigations into their infection, oncogenesis, and immune responses that protect from the associated tumors have been hampered by the exclusive tropism of these pathogens for humans. Mice with reconstituted human immune system components (HIS mice) provide the unique opportunity to study persistent infection, virus associated lymphoma formation, and cell-mediated immune control of EBV and KSHV. Moreover, since these pathogens are unique stimuli for cytotoxic human lymphocyte responses, they also allow us to characterize long-lasting cell-mediated immune control and the requirements for its initiation, which would also be desirable to achieve during antitumor vaccination in general. Thus, human γ-herpesvirus infection of HIS mice provides unique insights into the biology of these important human pathogens and human cell-mediated immune responses that are considered to be the main protective entity against tumors.

## Introduction

The two human γ-herpesviruses Epstein–Barr virus (EBV or HHV4) and Kaposi sarcoma-associated herpesvirus (KSHV or HHV8) are WHO class I carcinogens and responsible for around 10% of the infection-associated tumors in humans (1, 2). Even so they belong to the same subfamily of herpesviruses, their penetration of the human population, oncogenicity, and tissue tropism is quite different. While EBV persistently infects more than 90% of the human adult population, KSHV seropositivity is quite rare in Europe and the USA (<10%), but approaches 50% in Africa (3). The endothelial cell-derived Kaposi sarcoma is the only malignancy that is consistently associated with KSHV alone. In addition, KSHV is found in the lymphoproliferation multicentric Castleman’s disease, which can progress to non-Hodgkin’s lymphoma in the minority of cases (4), and primary effusion lymphoma (PEL) which in 90% of cases also harbors EBV (5). In addition to PELs, EBV is also found in various lymphocyte and epithelial cell malignancies, including Burkitt’s lymphoma, Hodgkin’s lymphoma, diffuse large B cell lymphoma (DLBCL), natural killer (NK)/T cell lymphoma, nasopharyngeal carcinoma, and gastric carcinoma (5). As already suggested by the breadth of tumors that it is associated with, EBV is also the much more growth-transforming virus of the two, readily immortalizing human B cells into lymphoblastoid cell lines (LCLs) upon infection *in vitro*. Furthermore, EBV is associated with so many different malignancies, because it adjusts its gene expression pattern to the differentiation stages of its main host cell, the human B cell, and thereby contributes to various degrees to the transformation in these different malignancies (6). The latent infection program with the largest number of expressed proteins is called latency III and is found in naïve B cells of healthy EBV carriers and DLBCL as well as LCL (7). During latency III, six nuclear proteins (EBNAs), two latent membrane proteins (LMPs), and non-translated miRNAs as well as EBERs are expressed. In latency II of Hodgkin’s lymphoma and germinal center B cells of healthy EBV carriers only EBNA1, the two LMPs and the non-translated RNAs are expressed. Finally, in latency I of Burkitt’s lymphoma and homeostatically dividing memory B cells, only EBNA1 and the non-translated RNAs are expressed. EBV is thought to persist in resting memory B cells without latent protein expression, only transcribing the non-translated RNAs from episomal viral DNA (8). Cognate antigen recognition by the B cell receptor is then able to reactivate EBV from this memory pool, and plasma cell differentiation is associated with lytic infectious virus production (9). Such lytic EBV infection in mucosal epithelia amplifies viral titers once more for shedding into saliva and transmission (10). In contrast to these distinct EBV infection programs, KSHV gene expression does not seem to be primarily restricted to the latency gene products latency-associated nuclear antigen, viral FLICE inhibitory protein (vFLIP), and viral D-type cyclin (vCyclin) in tumor tissues (5). Instead, expression of the lytic gene products K1, K2, and K15 seem to support the anti-apoptotic function of vFLIP to ensure survival of KSHV-associated tumor cells, which proliferate in part due to vCyclin expression (11). KSHV is thought to persist in long-lived plasma cells (12). How these patterns of viral oncogene expression are coordinated to cause KSHV- and EBV-associated pathogenesis and which immune compartments prevent them in healthy carriers of these human γ-herpesviruses has been difficult to study due to the exclusive tropism of these viruses for humans. With the advent of mice with reconstituted human immune system components (HIS mice), some of these questions can be addressed, and this review summarizes the insights into the fascinating biology of these human tumor viruses that could be gained so far.

## EBV and KSHV infection

Epstein–Barr virus was one of the first pathogens that HIS mice were challenged with (13–17). All programs of EBV infection in B cells were found after intraperitoneal infection of reconstituted NOD-*scid* $\gamma_{\text{c}}^{-/-}$ (NSG), NOD-*scid* $\gamma_{\text{c}}^{\text{truncated}}$ (NOG), BALB/c Rag2^−/−^ $\gamma_{\text{c}}^{-/-}$ (BRG), and human fetal liver plus human fetal thymus transplanted NOD-*scid* (BLT) mice, but latency III predominates (18, 19). Most of these studies found persistence of EBV in HIS mice for several months with circulating total viral loads in the blood of 10^4^ and 10^3^/ml in the serum after 4–5 weeks of infection with 10^5^ viral particles (20, 21). At this time point, total viral loads reach 10^7^ viral DNA copies/g in secondary lymphoid tissues like spleen and lymph nodes. These viral loads are comparable to blood viral loads in patients with symptomatic primary EBV infection, called infectious mononucleosis (IM) (22) that surprisingly do not differ very much from overall blood viral loads of asymptomatic primary infection (23, 24). In most of these studies, the B95-8 EBV strain was used, which reactivates only very weakly into lytic replication and was originally isolated from an American IM patient (25, 26). Indeed, in a direct comparison of wild-type (wt) and BZLF1-deficient (ZKO) EBV viruses on the B95-8 background viral titer differences were only observed at week three after infection (20). At this time point, some wt EBV-infected HIS mice reached already 10^4^ DNA copies/ml in the blood, while ZKO EBV-infected mice have 10^3^. These characteristics can be altered by using different viral strains for HIS mouse infection. Infection with 10^5^ B cell infectious particles of the M81 EBV strain, which was isolated from a Chinese nasopharyngeal carcinoma patient, leads to 10^5^–10^6^ DNA copies/ml in the peripheral blood of HIS mice after 4–5 weeks of infection (27), and other EBV strains fall in between the two extremes of B95-8 and M81 (28). Thus, EBV infection with 10^5^ infectious viral particles causes a primary EBV infection in HIS mice with similar viral loads that have been reported in human symptomatic and asymptomatic primary infections that can persist for months, even so many HIS mice with such high-persistent viral loads succumb to EBV-induced lymphoproliferations, as discussed below.

Kaposi sarcoma-associated herpesvirus infection of HIS mice on its own is a transient phenomenon with less than 20% of mice maintaining KSHV after infection with 10^5^–10^7^ infectious particles at 5 weeks post infection (29). However, repeated infections can maintain KSHV for several months in BLT mice on the NSG mouse background, as assessed by expression of KSHV gene products and KSHV-encoded GFP 2 weeks after the final inoculation (30). However, co-infection with EBV maintains KSHV in the majority of infected HIS mice of the NSG mouse background after single infection (29). During both transient and persistent KSHV infections, the virus can be found in human B cells (29, 30), and after 5 weeks of double-infection of KSHV with EBV, KSHV is primarily observed in EBV-infected B cells (29). Double-infection leads to 25% mortality of HIS mice after 5 weeks of infection, while single EBV infection causes much less pathology (29). These findings suggest that HIS mice can serve as *in vivo* infection models for both of these oncogenic γ-herpesviruses and that KSHV, surprisingly, relies on EBV for persistence in this model.

## EBV and KSHV Tumorigenesis

**Figure 1:**
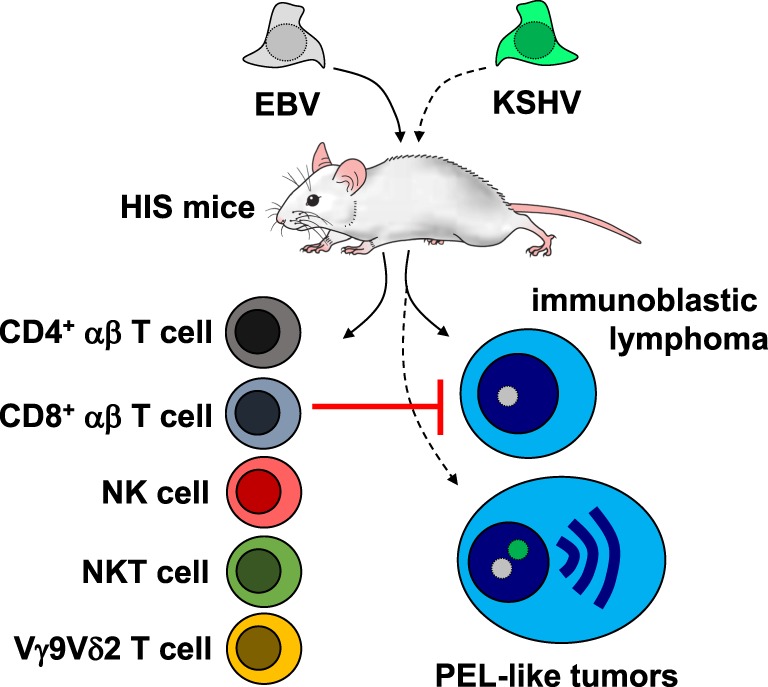
Tumorigenesis and immune control of Epstein–Barr virus (EBV) with and without Kaposi sarcoma-associated herpesvirus (KSHV) co-infection in mice with human immune system components (HIS mice). KSHV co-infection converts EBV-induced immunoblastic lymphoma into primary effusion lymphoma (PEL)-like tumors. EBV-associated immunoblastic lymphomas are restricted by cytotoxic lymphocytes in humanized mice, including CD4^+^ and CD8^+^ T cells, natural killer (NK) cells, NKT cells, and Vγ9Vδ2 T cells.

The above-discussed mortality is probably in part connected to the lymphomagenesis that can be observed in HIS mice after single EBV and EBV plus KSHV co-infection. After 5–6 weeks of infection with 10^5^ infectious particles of the B95-8 EBV, 20–30% of mice develop macroscopically visible tumors in various organs, including spleen, pancreas, kidney, liver, and lymph nodes (16, 20, 21). Tumor incidence does not seem to be significantly different in EBV-infected BLT mice (18). These are EBV latency III B cell tumors, which can be grown as EBV-transformed B cell lines *in vitro* after dissociation of the visible tumors (Figure 1) (16, 29, 31). The ability of HIS mice to develop B cell lymphomas has been used to query the role of different latent EBV antigens and lytic EBV replication in EBV-associated lymphomagenesis. Along these lines, the nuclear antigen 3B of EBV (EBNA3B) has been found to be deleted in a subset of EBV-associated DLBCLs in patients (31, 32). Accordingly, EBNA3B-deficient B95-8 EBV causes macroscopically visible tumors in 50% of HIS mice after 4 weeks of infection (31). These tumors are, interestingly, devoid of T cell infiltrates and transcriptome analysis of EBNA3B-deficient EBV-transformed B cell lines that were derived from tumors in HIS mice, and DLBCL patients demonstrated a loss of pro-inflammatory chemokine production (31). Restoration of CXCL10 expression in EBNA3A-deficient tumor cell lines resulted in T cell-mediated immune control *in vivo*. In addition, the transcriptome analysis revealed that EBNA3B-deficient EBV-transformed B cells of HIS mice were more similar to patient-derived DLBCL cell lines in their gene expression than LCLs that had been transformed with EBNA3B-deficient EBV *in vitro* (31). These findings established EBNA3B as a viral tumor suppressor by its control over pro-inflammatory chemokines.

Furthermore, it was noted that loss of lytic EBV replication decreased the ability of infection to cause lymphomagenesis (18). This at first sight counterintuitive behavior, namely that cell-destructive lytic EBV infection should benefit B cell transformation and lymphoma growth, was suggested to result from a pro-inflammatory environment upon early, possibly abortive lytic EBV replication, but the responsible pro-inflammatory components have not been identified so far. Nevertheless, decreased lymphomagenesis by the B95-8 EBV virus that lacks the immediate early lytic transactivator BZLF1 was also observed in a second study (20), and the BZLF1 overexpressing virus induced the same amount of lymphomas, but these contained up to 30% of early, but not late lytic EBV antigen expression (33), confirming a possible role of abortive lytic replication in lymphomagenesis by EBV.

In the same way, KSHV co-infection with EBV increases lytic EBV replication and EBV-associated tumorigenesis (29). Interestingly, in this first small animal *in vivo* model of KSHV persistence, the developing tumors carry KSHV in one-third of EBV-infected lymphoma cells. This leads to an upregulation of gene expression that is associated with plasma cell differentiation, including the plasma cell fingerprint that is characteristic for PELs (Figure 1) (34). About 39% of KSHV and EBV double-infected mice with PEL-like tumors succumb to their disease after 1 month (29), while 25% of patients with PEL succumb to tumor progression within 4 months (35). Therefore, KSHV and EBV double-infection that leads to PEL formation causes significant mortality. Interestingly, double-infection of KSHV with the lytic EBV replication-deficient BZLF1 knockout strain of B95-8 abolishes the gain of lymphomagenesis upon infection with both viruses (29). Furthermore, early and late lytic EBV gene expression were observed at higher frequencies in KSHV and EBV double-infected lymphomas of patients than in a heterogenous groups of EBV single-infected lymphomas. In good agreement, lytic EBV replication inhibition with ganciclovir caused complete sustained PEL remission in a patient with EBV and KSHV double-positive lymphoma (36), but only transient improvement in a patient with KSHV single-positive PEL (37). Thus, HIS mice infections with EBV alone and KSHV co-infection have revealed an unexpected role for lytic EBV replication during virus-associated lymphomagenesis, which might be even diagnostically useful to predict the risk of EBV-associated malignancy development during immune suppression (38).

## EBV- and KSHV-Specific Immune Control

Primary immunodeficiencies that predispose for EBV-associated pathologies point toward an essential role for cytotoxic lymphocytes in the immune control of this oncogenic γ-herpesvirus (39, 40). The respective mutations affect the perforin degranulation machinery, co-stimulatory receptors on cytotoxic lymphocytes and DNA binding proteins that are required for the differentiation of NK, NKT, γδT, and CD8^+^ αβ T cells. Much less is known about the protective immune responses against KSHV in humans, but the available information points to similar requirements as in the immune control of EBV (41).

Some of these cytotoxic lymphocyte compartments have been interrogated during EBV infection of HIS mice. These studies initially focused on T cell responses. In loss-of-function experiments, antibody-mediated depletion of all T cells or CD8^+^ and CD4^+^ T cells was found individually to increase EBV viral loads and associated lymphomagenesis upon infection (Figure 1) (16, 33, 42). Blocking of the co-stimulatory 2B4 receptor, which is compromised in one primary immunodeficiency (Duncan disease or XLP1) that predisposes for uncontrolled EBV infection, resulted in the loss of CD8^+^ T cell-mediated immune control and elevated viral loads as well as increased tumor frequency (43). In gain-of-function experiments, adoptive transfer of lytic EBV antigen-specific CD8^+^ T cells was able to further reduce the low level of lytic EBV replication upon B95-8 infection of HIS mice (20). Furthermore, late lytic EBV antigen and LCL differentiation-specific CD4^+^ T cells were able to lower viral loads in EBV-infected HIS mice (44). If human immune system reconstitution is performed by unseparated cord blood injection rather than differentiation from human hematopoietic progenitor cells, the established T cell compartment rather supports EBV-associated lymphomagenesis, even in the absence of viral oncogenes (45, 46). These cord blood T cells provide CD4^+^ T cell help for EBV-associated lymphomas (45). This T cell help can, however, be converted into immune control by antibody-mediated blocking of the inhibitory receptors PD-1 and CTLA-4 (47), presumably mimicking a T cell compartment that might resemble EBV-associated Hodgkin’s lymphoma, a tumor entity that can be efficiently treated by check-point blockade immunotherapy (48). Thus, T cell-mediated immune control of EBV can be interrogated in HIS mice, and depending on the method of immune compartment reconstitution, immune compartments of healthy EBV carriers or patients with EBV-associated malignancies can be modeled.

In addition, innate lymphocyte compartments have also been interrogated for their contribution to immune control of EBV. NK cell depletion leads to elevated viral loads and tumor formation in EBV-infected HIS mice (Figure 1) (21, 49). Lytic EBV infection is primarily controlled by the early-differentiated NK cells of HIS mice, because infection with BZLF1 knockout EBV is not affected by NK cell depletion. These early-differentiated NK cells also expand in children with IM (22). It seems that further differentiated NK cells with HLA-haplotype-specific inhibitory receptors can be recruited to this response in mixed HLA-mismatched hematopoietic progenitor cell reconstitutions, which presumably allow allogeneic recognition of EBV-infected B cells of one donor by the further differentiation NK cells of the other donor (49). In addition to NK cells, adoptive transfer of CD8^+^ NKT and Vγ9Vδ2 T cells restricts EBV-associated lymphomas in HIS mice (Figure 1) (50, 51). Furthermore, Vγ9Vδ2 T cell activation with phosphoantigens results in improved immune control of successive EBV infection in HIS mice (52). Interestingly, innate and adaptive lymphocyte compartments seem to compensate each other, because loss of NK cell-mediated immune control leads to enhanced CD8^+^ T cell expansion during EBV infection of HIS mice. It will be interesting to elucidate which EBV infection programs are controlled by these different lymphocyte populations and which receptors on NK, NKT, and γδ T cells mediate EBV restriction *in vivo*. Stimulation of these cytotoxic lymphocyte compartments by vaccination could correct loss of EBV-specific immune control in patients with EBV-associated malignancies, but also teach us how to induce comprehensive cell-mediated immune control against tumors in general.

## Conclusion and Outlook

While we are beginning to understand the protective lymphocyte compartments during EBV infection, their characterization for KSHV infection is in its infancy. Furthermore, we still have an incomplete understanding of how the comprehensive immune control by cytotoxic lymphocytes against EBV is initiated; even so, EBV is the prototypic viral pathogen that elicits CD8^+^ T cell lymphocytosis during symptomatic infection in IM patients. A detailed understanding of the characteristics of a comprehensive immune control by cytotoxic lymphocytes and the mechanisms that lead to its priming should guide us to develop vaccines to elicit such immune control, not only against EBV in patients with associated malignancies, but also tumors or badly controlled viral infections in general.

## Author Contributions

The author has no financial conflicts of interest with the subject discussed in the manuscript. He has planned and written the paper.

## Conflict of Interest Statement

The author declares that the research was conducted in the absence of any commercial or financial relationships that could be construed as a potential conflict of interest.

## References

[1] Bouvard V Baan R Straif K Grosse Y Secretan B El Ghissassi F A review of human carcinogens – part B: biological agents. Lancet Oncol (2009) 10(4):321–2. 10.1016/S1470-2045(09)70096-8 19350698

[2] Parkin DM. The global health burden of infection-associated cancers in the year 2002. Int J Cancer (2006) 118(12):3030–44. 10.1002/ijc.21731 16404738

[3] Mesri EA Cesarman E Boshoff C. Kaposi’s sarcoma and its associated herpesvirus. Nat Rev Cancer (2010) 10(10):707–19. 10.1038/nrc2888 20865011 PMC4721662

[4] Oksenhendler E Boulanger E Galicier L Du MQ Dupin N Diss TC High incidence of Kaposi sarcoma-associated herpesvirus-related non-Hodgkin lymphoma in patients with HIV infection and multicentric Castleman disease. Blood (2002) 99(7):2331–6. 10.1182/blood.V99.7.2331 11895764

[5] Cesarman E. Gammaherpesviruses and lymphoproliferative disorders. Annu Rev Pathol (2014) 9:349–72. 10.1146/annurev-pathol-012513-104656 24111911

[6] Thorley-Lawson DA Gross A Persistence of the Epstein-Barr virus and the origins of associated lymphomas. N Engl J Med (2004) 350(13):1328–37. 10.1056/NEJMra032015 15044644

[7] Babcock JG Hochberg D Thorley-Lawson AD. The expression pattern of Epstein-Barr virus latent genes in vivo is dependent upon the differentiation stage of the infected B cell. Immunity (2000) 13(4):497–506. 10.1016/S1074-7613(00)00049-2 11070168

[8] Babcock GJ Decker LL Volk M Thorley-Lawson DA. EBV persistence in memory B cells in vivo. Immunity (1998) 9(3):395–404. 10.1016/S1074-7613(00)80622-6 9768759

[9] Laichalk LL Thorley-Lawson DA. Terminal differentiation into plasma cells initiates the replicative cycle of Epstein-Barr virus in vivo. J Virol (2005) 79(2):1296–307. 10.1128/JVI.79.2.1296-1307.2005 15613356 PMC538585

[10] Hutt-Fletcher LM The long and complicated relationship between Epstein-Barr virus and epithelial cells. J Virol (2017) 91(1):e01677-16. 10.1128/JVI.01677-16 27795426 PMC5165189

[11] Martin DF Kuppermann BD Wolitz RA Palestine AG Li H Robinson CA. Oral ganciclovir for patients with cytomegalovirus retinitis treated with a ganciclovir implant. Roche Ganciclovir Study Group. N Engl J Med (1999) 340(14):1063–70. 10.1056/NEJM199904083401402 10194235

[12] Ganem D. KSHV infection and the pathogenesis of Kaposi’s sarcoma. Annu Rev Pathol (2006) 1:273–96. 10.1146/annurev.pathol.1.110304.100133 18039116

[13] Traggiai E Chicha L Mazzucchelli L Bronz L Piffaretti JC Lanzavecchia A Development of a human adaptive immune system in cord blood cell-transplanted mice. Science (2004) 304(5667):104–7. 10.1126/science.1093933 15064419

[14] Melkus MW Estes JD Padgett-Thomas A Gatlin J Denton PW Othieno FA Humanized mice mount specific adaptive and innate immune responses to EBV and TSST-1. Nat Med (2006) 12(11):1316–22. 10.1038/nm1431 17057712

[15] Yajima M Imadome K Nakagawa A Watanabe S Terashima K Nakamura H A new humanized mouse model of Epstein-Barr virus infection that reproduces persistent infection, lymphoproliferative disorder, and cell-mediated and humoral immune responses. J Infect Dis (2008) 198(5):673–82. 10.1086/590502 18627269

[16] Strowig T Gurer C Ploss A Liu YF Arrey F Sashihara J Priming of protective T cell responses against virus-induced tumors in mice with human immune system components. J Exp Med (2009) 206(6):1423–34. 10.1084/jem.20081720 19487422 PMC2715061

[17] Shultz LD Saito Y Najima Y Tanaka S Ochi T Tomizawa M Generation of functional human T-cell subsets with HLA-restricted immune responses in HLA class I expressing NOD/SCID/IL2r gamma^null^ humanized mice. Proc Natl Acad Sci U S A (2010) 107(29):13022–7. 10.1073/pnas.1000475107 20615947 PMC2919921

[18] Ma SD Hegde S Young KH Sullivan R Rajesh D Zhou Y A new model of Epstein-Barr virus infection reveals an important role for early lytic viral protein expression in the development of lymphomas. J Virol (2011) 85(1):165–77. 10.1128/JVI.01512-10 20980506 PMC3014199

[19] Cocco M Bellan C Tussiwand R Corti D Traggiai E Lazzi S CD34^+^ cord blood cell-transplanted Rag2^-/-^ gamma_c_^-/-^ mice as a model for Epstein-Barr virus infection. Am J Pathol (2008) 173(5):1369–78. 10.2353/ajpath.2008.071186 18845836 PMC2570127

[20] Antsiferova O Müller A Rämer P Chijioke O Chatterjee B Raykova A Adoptive transfer of EBV specific CD8^+^ T cell clones can transiently control EBV infection in humanized mice. PLoS Pathog (2014) 10(8):e1004333. 10.1371/journal.ppat.1004333 25165855 PMC4148450

[21] Chijioke O Muller A Feederle R Barros MH Krieg C Emmel V Human natural killer cells prevent infectious mononucleosis features by targeting lytic Epstein-Barr virus infection. Cell Rep (2013) 5(6):1489–98. 10.1016/j.celrep.2013.11.041 24360958 PMC3895765

[22] Azzi T Lunemann A Murer A Ueda S Beziat V Malmberg KJ Role for early-differentiated natural killer cells in infectious mononucleosis. Blood (2014) 124(16):2533–43. 10.1182/blood-2014-01-553024 25205117 PMC4199955

[23] Jayasooriya S de Silva TI Njie-jobe J Sanyang C Leese AM Bell AI Early virological and immunological events in asymptomatic Epstein-Barr virus infection in African children. PLoS Pathog (2015) 11(3):e1004746. 10.1371/journal.ppat.1004746 25816224 PMC4376400

[24] Abbott RJ Pachnio A Pedroza-Pacheco I Leese AM Begum J Long HM Asymptomatic primary infection with Epstein-Barr virus: observations on young adult cases. J Virol (2017) 91(21):e00382-17. 10.1128/JVI.00382-17. 28835490 PMC5640854

[25] Miller G Lipman M. Release of infectious Epstein-Barr virus by transformed marmoset leukocytes. Proc Natl Acad Sci U S A (1973) 70(1):190–4. 10.1073/pnas.70.1.190 4346033 PMC433213

[26] Miller G Lipman M. Comparison of the yield of infectious virus from clones of human and simian lymphoblastoid lines transformed by Epstein-Barr virus. J Exp Med (1973) 138(6):1398–412. 10.1084/jem.138.6.1398 4357683 PMC2139465

[27] Tsai MH Raykova A Klinke O Bernhardt K Gartner K Leung CS Spontaneous lytic replication and epitheliotropism define an Epstein-Barr virus strain found in carcinomas. Cell Rep (2013) 5(2):458–70. 10.1016/j.celrep.2013.09.012 24120866

[28] Tsai MH Lin X Shumilov A Bernhardt K Feederle R Poirey R The biological properties of different Epstein-Barr virus strains explain their association with various types of cancers. Oncotarget (2017) 8(6):10238–54. 10.18632/oncotarget.14380 28052012 PMC5354655

[29] McHugh D Caduff N Barros MHM Rämer P Raykova A Murer A Persistent KSHV infection increases EBV-associated tumor formation in vivo via enhanced EBV lytic gene expression. Cell Host Microbe (2017) 22(1):61–73. 10.1016/j.chom.2017.06.009 28704654

[30] Wang LX Kang G Kumar P Lu W Li Y Zhou Y Humanized-BLT mouse model of Kaposi’s sarcoma-associated herpesvirus infection. Proc Natl Acad Sci U S A (2014) 111(8):3146–51. 10.1073/pnas.1318175111 24516154 PMC3939909

[31] White RE Ramer PC Naresh KN Meixlsperger S Pinaud L Rooney C EBNA3B-deficient EBV promotes B cell lymphomagenesis in humanized mice and is found in human tumors. J Clin Invest (2012) 122(4):1487–502. 10.1172/JCI58092 22406538 PMC3314448

[32] Gottschalk S Ng CY Perez M Smith CA Sample C Brenner MK An Epstein-Barr virus deletion mutant associated with fatal lymphoproliferative disease unresponsive to therapy with virus-specific CTLs. Blood (2001) 97(4):835–43. 10.1182/blood.V97.4.835 11159505

[33] Ma SD Yu X Mertz JE Gumperz JE Reinheim E Zhou Y An Epstein-Barr virus (EBV) mutant with enhanced BZLF1 expression causes lymphomas with abortive lytic EBV infection in a humanized mouse model. J Virol (2012) 86(15):7976–87. 10.1128/JVI.00770-12 22623780 PMC3421695

[34] Klein U Gloghini A Gaidano G Chadburn A Cesarman E Dalla-Favera R Gene expression profile analysis of AIDS-related primary effusion lymphoma (PEL) suggests a plasmablastic derivation and identifies PEL-specific transcripts. Blood (2003) 101(10):4115–21. 10.1182/blood-2002-10-3090 12531789

[35] El-Fattah MA. Clinical characteristics and survival outcome of primary effusion lymphoma: a review of 105 patients. Hematol Oncol (2017) 35(4):878–83. 10.1002/hon.2372 27859456

[36] Pereira R Carvalho J Patricio C Farinha P. Sustained complete remission of primary effusion lymphoma with adjunctive ganciclovir treatment in an HIV-positive patient. BMJ Case Rep (2014) 2014:bcr2014204533. 10.1136/bcr-2014-204533 25312890 PMC4195214

[37] Ozbalak M Tokatli I Ozdemirli M Tecimer T Ar MC Ornek S Is valganciclovir really effective in primary effusion lymphoma: case report of an HIV^-^ EBV^-^ HHV8^+^ patient. Eur J Haematol (2013) 91(5):467–9. 10.1111/ejh.12174 23865480

[38] Kanakry J Ambinder R. The biology and clinical utility of EBV monitoring in blood. Curr Top Microbiol Immunol (2015) 391:475–99. 10.1007/978-3-319-22834-1_17 26428386

[39] Cohen JI. Primary immunodeficiencies associated with EBV disease. Curr Top Microbiol Immunol (2015) 390(Pt 1):241–65. 10.1007/978-3-319-22822-8_10 26424649 PMC6349415

[40] Tangye SG Palendira U Edwards ES. Human immunity against EBV-lessons from the clinic. J Exp Med (2017) 214(2):269–83. 10.1084/jem.20161846 28108590 PMC5294862

[41] Pasic S Cupic M Lazarevic I. HHV-8-related hemophagocytic lymphohistiocytosis in a boy with XLP phenotype. J Pediatr Hematol Oncol (2012) 34(6):467–71. 10.1097/MPH.0b013e3182375372 22258354

[42] Yajima M Imadome K Nakagawa A Watanabe S Terashima K Nakamura H T cell-mediated control of Epstein-Barr virus infection in humanized mice. J Infect Dis (2009) 200(10):1611–5. 10.1086/644644 19832115

[43] Chijioke O Marcenaro E Moretta A Capaul R Munz C The SAP-dependent 2B4 receptor mediates CD8^+^ T cell dependent immune control of Epstein Barr virus infection in mice with reconstituted human immune system components. J Infect Dis (2015) 212(5):803–7. 10.1093/infdis/jiv114 25722295

[44] Linnerbauer S Behrends U Adhikary D Witter K Bornkamm GW Mautner J. Virus and autoantigen-specific CD4^+^ T cells are key effectors in a SCID mouse model of EBV-associated post-transplant lymphoproliferative disorders. PLoS Pathog (2014) 10(5):e1004068. 10.1371/journal.ppat.1004068 24853673 PMC4031221

[45] Ma SD Xu X Plowshay J Ranheim EA Burlingham WJ Jensen JL LMP1-deficient Epstein-Barr virus mutant requires T cells for lymphomagenesis. J Clin Invest (2015) 125(1):304–15. 10.1172/JCI76357 25485679 PMC4382240

[46] Ma SD Tsai MH Romero-Masters JC Ranheim EA Huebner SM Bristol J LMP1 and LMP2A collaborate to promote Epstein-Barr virus (EBV)-induced B cell lymphomas in a cord blood-humanized mouse model but are not essential. J Virol (2017) 91(7):e01928-16. 10.1128/JVI.01928-16 28077657 PMC5355617

[47] Ma SD Xu X Jones R Delecluse HJ Zumwalde NA Sharma A PD-1/CTLA-4 blockade inhibits Epstein-Barr virus-induced lymphoma growth in a cord blood humanized-mouse model. PLoS Pathog (2016) 12(5):e1005642. 10.1371/journal.ppat.1005642 27186886 PMC4871349

[48] Ansell SM Lesokhin AM Borrello I Halwani A Scott EC Gutierrez M PD-1 blockade with nivolumab in relapsed or refractory Hodgkin’s lymphoma. N Engl J Med (2015) 372(4):311–9. 10.1056/NEJMoa1411087 25482239 PMC4348009

[49] Landtwing V Raykova A Pezzino G Beziat V Marcenaro E Graf C Cognate HLA absence in trans diminishes human NK cell education. J Clin Invest (2016) 126(10):3772–82. 10.1172/JCI86923 27571408 PMC5096830

[50] Yuling H Ruijing X Li L Xiang J Rui Z Yujuan W EBV-induced human CD8^+^ NKT cells suppress tumorigenesis by EBV-associated malignancies. Cancer Res (2009) 69(20):7935–44. 10.1158/0008-5472.CAN-09-0828 19808969

[51] Zumwalde NA Sharma A Xu X Ma S Schneider CL Romero-Masters JC Adoptively transferred Vgamma9Vdelta2 T cells show potent antitumor effects in a preclinical B cell lymphomagenesis model. JCI Insight (2017) 2(13): e93179 10.1172/jci.insight.93179 PMC5499361 28679955

[52] Xiang Z Liu Y Zheng J Liu M Lv A Gao Y Targeted activation of human Vgamma9Vdelta2-T cells controls Epstein-Barr virus-induced B cell lymphoproliferative disease. Cancer Cell (2014) 26(4):565–76. 10.1016/j.ccr.2014.07.026 25220446

